# Worldwide surveillance of self-reported sitting time: a scoping review

**DOI:** 10.1186/s12966-020-01008-4

**Published:** 2020-09-03

**Authors:** M. Mclaughlin, A. J. Atkin, L. Starr, A. Hall, L. Wolfenden, R. Sutherland, J. Wiggers, A. Ramirez, P. Hallal, M. Pratt, B. M. Lynch, K. Wijndaele, on behalf of the Sedentary Behaviour Council Global Monitoring Initiative Working Group, Saiful Adli, Paul A. Gardiner, Ciaran B. Doyle, Angela Meadows, Ruth M. Mabry, Alberto Florez Pregonero, Kabir P. Sadarangani, Nyssa T. Hadgraft, Terry Boyle, Nicolas Aguilar Farias, Jacqueline L. Mair, Siosaia F. Hafoka, Gregore Iven Mielke, Selina Khoo Phaik Lin, Vienna R. McLeod, Chathuranga Ranasinghe, Paul C. Storning, Ing-Mari Dohrn, Falk Müller-Riemenschnieder, Lyutha Al Subhi, Anne Chu Hin Yee, Mayuri Gad, Adilson Marques, Elli Kontostoli

**Affiliations:** 1 School of Medicine and Public Health, University of Newcastle, Newcastle, 2308 Australia; 2 Hunter New England Population Health, Locked Bag 10, Wallsend, NSW 2287 Australia; 3 Hunter Medical Research Institute, Newcastle, NSW 2300 Australia; 4 Priority Research Centre in Health Behaviour, University of Newcastle, Newcastle, NSW Australia; 5 Faculty of Medicine and Health Sciences, University of East Anglia, Norwich, UK; 6 Warwick Medical School, University of Warwick, Coventry, West Midlands UK; 7 School of Medicine, Los Andes University, Bogotá, Colombia; 8 University of California San Diego, San Diego, California USA; 9 Cancer Epidemiology Division, Cancer Council Victoria, Melbourne, Victoria Australia; 10 Centre for Epidemiology and Biostatistics, Melbourne School of Population and Global Health, The University of Melbourne, Melbourne, Victoria Australia; 11 MRC Epidemiology Unit, University of Cambridge School of Clinical Medicine, Cambridge, Cambridgeshire UK

**Keywords:** Sitting time, Sedentary behaviour, Surveillance

## Abstract

**Background:**

Prolonged sitting time is a risk factor for chronic disease, yet recent global surveillance is not well described. The aims were to clarify: (i) the countries that have collected country-level data on self-reported sitting time; (ii) the single-item tools used to collect these data; and (iii) the duration of sitting time reported across low- to high-income countries.

**Methods:**

Country-level data collected within the last 10 years using single-item self-report were included. The six-stage methodology: (1) reviewing Global Observatory for Physical Activity! Country Cards; (2–4) country-specific searches of PubMed, the Demographic and Health Survey website and Google; (5) analysing the Eurobarometer 88.4; and (6) country-specific searches for World Health Organization STEPwise reports.

**Results:**

A total of 7641 records were identified and screened for eligibility. Sixty-two countries (29%) reported sitting time representing 47% of the global adult population. The majority of data were from high-income (61%) and middle income (29%) countries. The tools used were the International Physical Activity Questionnaire (IPAQ; *n* = 34), a modified IPAQ (*n* = 1) or the Global Physical Activity Questionnaire (GPAQ; *n* = 27). The median of mean daily sitting times was 4.7 (IQR: 3.5–5.1) hours across all countries. Higher-income countries recorded a longer duration of sitting time than lower-income countries (4.9 vs 2.7 h).

**Conclusions:**

This study provides an updated collation of countries collecting self-reported sitting time data. The daily sitting time findings should be interpreted cautiously. Current surveillance of sitting time is limited by a lack of coverage. Measures of population sitting time that are valid, feasible and sensitive to change should be embedded within global surveillance systems, to help guide future policy, research and practice.

**Trial registration:**

Not applicable.

## Background

Sedentary behaviour is characterised as any waking behaviour at an intensity ≤1.5 METs in a sitting or reclining posture [1]. Sitting time is a sub-component of sedentary behaviour and a common measure of sedentary behaviour [2]. Sedentary behaviour is associated with a range of adverse health outcomes, including, but not limited to: all-cause, cardiovascular and cancer mortality, type 2 diabetes and depression [3–10]. In particular, the combination of high amounts of sedentary behaviour and low amounts of moderate-to-vigorous physical activity is associated with all-cause and cardiovascular mortality [3, 7], As such, the World Health Organization is currently in the process of revising the 2010 global physical activity guidelines to include recommendations related to sedentary behaviour across all age groups [11].

Public health surveillance systems are used to identify emerging health threats, monitor changes in health and risk factors, guide programs to target threats and prioritise public health action [12]. Global surveillance data enables cross-country comparisons, and can be used to assess the influence of national policy initiatives on health risks and diseases [12, 13]. Such systems are recommended by the United Nations and World Health Organization and are increasingly being applied to non-communicable disease risks [14, 15]. Good surveillance systems can provide comparable actionable data and are characterised by valid, low cost and feasible assessments of risk in populations [12].

Sitting time is a common measure of sedentary behaviour [2]. Device-based measures of sitting time are more valid than self-reported sitting time, but are high cost, burdensome for participants and not yet widely used [2, 16]. Although people tend to under-report sitting time (1.4–2.1 h less than device-based), there are several self-report measures that have evidence of reliability and validity [2, 16–22] providing a potentially low cost, feasible option for use in national surveillance systems [14]. One promising method is to measure how long seated activities are undertaken (e.g. time spent driving, watching television), rather than asking how long someone has spent sitting [23]. However, single-item measurement of total sitting time may remain important for population surveillance as it is highly feasible.

Given the adverse impacts of sedentary behaviour, it is important to measure this risk factor globally. Collated data on self-reported physical activity from 168 countries, collected within the last 20 years, are available [24]. However, a comprehensive collation of sedentary behaviour data collected within the last 10 years does not currently exist. To our knowledge, only one previous study has collated data across countries globally on self-reported sitting time [9]. Rezende et al. found that adults from across 54 countries sit on average 4.7 h/day (282 min/day, weighted mean by country population) [9]. They used data from 2002 to 2011 collected from three main sources (Eurobarometer, World Health Organisation STEPwise approach to Surveillance (STEPS) and the International Prevalence Study) [14, 25, 26]. They additionally searched scientific databases yielding only five additional sources [9]. Other studies have collated smaller data sets, for example a 20-country comparison [26] or European-only data [25, 27].

To inform future global surveillance of sedentary behaviour, this study addressed the following three key questions: (i) What countries have collected country-level data on self-reported sitting time? (ii) What single-item self-report tools have countries used to collect such data? (iii) What is the duration of self-report, country-level sitting time by low-, lower-middle, upper-middle and high-income countries?

## Methods

This scoping review is reported in accordance with the PRISMA-ScR checklist (Supplementary file 1) [28]. The methods described here were outlined in a methods manual [29], which was executed through the Global Sedentary Behaviour Monitoring Initiative. This initiative is led by the Sedentary Behaviour Council (SBC) and Global Observatory for Physical Activity (GoPA!) Council of the International Society for Physical Activity and Health (ISPAH). The search strategy of the Global Sedentary Behaviour Monitoring Initiative included other outcomes not reported here.

### Eligibility criteria

**Fig. 1:**
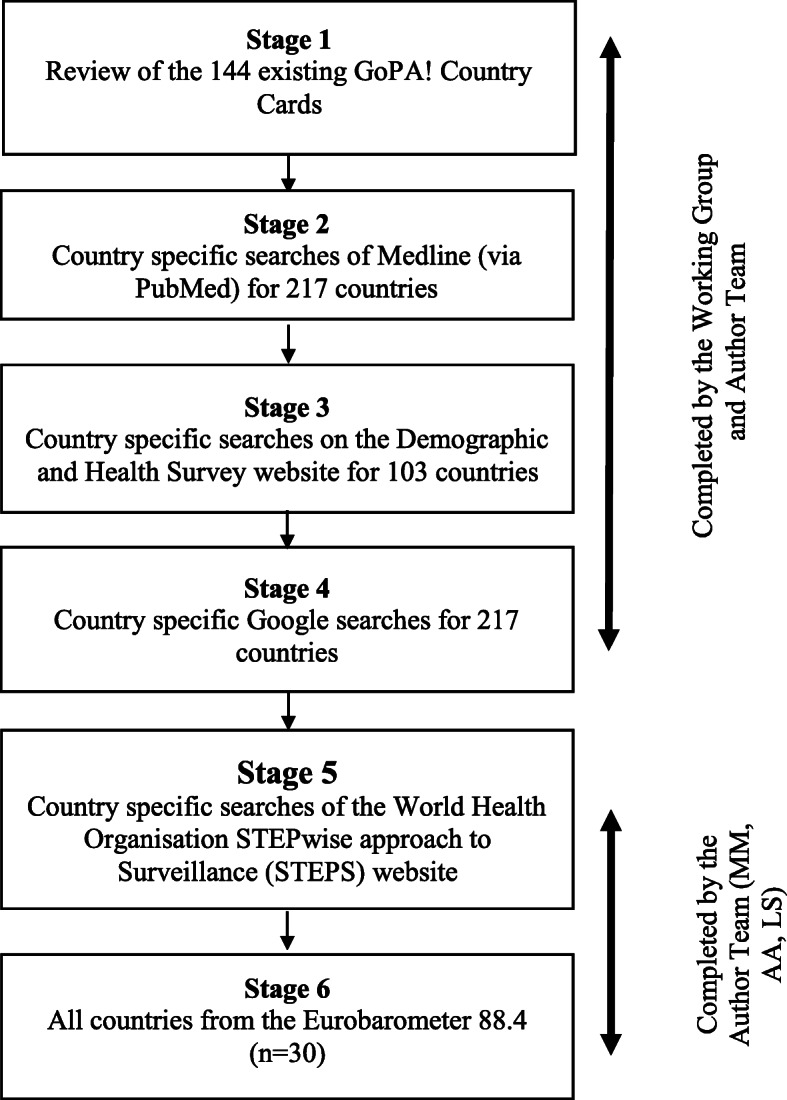
Search strategy

We excluded studies where data were collected more than 10 years before the initial search date (May 2018), to ensure the recency of data. We excluded studies limited to a single sex to improve data representativeness. We included studies in English. We additionally sought translations of reports in languages other than English for stage 5 of the search strategy (Fig. 1). We included both published and unpublished data from scientific and grey literature. The outcomes of interest for this report were: (i) countries reporting country-level data on self-reported sitting time collected in the last 10 years (2008–2018), in an adult population (age 15+); and (ii) the single-item self-report measure used to collect these data; (iii) the minutes of total daily sitting time reported within these data. We excluded data from multi-item self-report tools and device-measured sedentary behaviour, due to issues with harmonisation and limited availability of country-level data [2, 22].

### Classification of countries

In alignment with the GoPA! methodology applied to monitoring physical activity surveillance [30], we searched for data across 217 countries. Starting with a list of 215 countries derived from the World Bank [31], we subsequently split the United Kingdom into the four home nations (England, Scotland, Wales, and Northern Ireland), and combined information from China and Taiwan. For analyses, we classified countries by income level, using the 2020 World Bank’s classification [31]. We subsequently consolidated to 215 countries, as described in stage 6 below (Fig. 1).

### Search strategy

The search strategy consists of six stages (Fig. 1).

Stages 1–4 were completed by a Working Group and members of the Author team (MM, AR, AA) between May 2018 and December 2018. We recruited a Working Group of volunteers (*n* = 25) via email (March 2018) from the membership of the ISPAH Sedentary Behaviour Council. Each Working Group member was trained to conduct the searches through videoconferencing (April 2018) by one author (MM), and provided with a methods manual detailing the search strategy [29]. Throughout the search process, MM and AA assisted the Working Group to conduct searches via email and videoconferencing. Working Group members were allocated to countries (*n* = 9–14). Where possible, the Working Group members were allocated to countries from their region of residence, and surrounding countries.

#### Stage 1

The Working Group identified the data source cited for the physical activity prevalence estimate in each of “The 1st Physical Activity Almanac” GoPA! country cards (*n* = 139), as well as for five additional country cards added to the website since the launch of the first Almanac. These sources (*n* = 144) were then searched for relevant information on sitting time.

#### Stage 2

Country-specific searches of Medline were made through PubMed for each country, using the search terms listed in Supplementary file 2. All records were screened by the respective member of the Working Group.

#### Stage 3

The Demographic and Health Survey website contains data on health from country-level surveys. Country-specific searches were made for countries listed on its website (*n* = 105).

#### Stage 4

Seven country-specific Google searches were made for each country using the terms listed in Supplementary file 2, which were entered in ‘www.Google.com/ncr’ to avoid country redirect differences in country-specific versions of Google. The first 20 titles were reviewed for each respective search.

Additional studies were also recommended by the Working Group’s collective knowledge and via snowballing from identified studies.

### Stages 5–6

Three authors (MM, LS, AA) conducted stage 5–6.

#### Stage 5

The World Health Organisation STEPwise approach to Surveillance (STEPS) is a standardised framework of data collection for countries [14]. For all countries, country-specific searches of the World Health Organisation STEPwise approach to Surveillance (STEPS) website were conducted. Where reports were not in English, translations were sought.

#### Stage 6

The Eurobarometer is a periodical survey that takes place in European countries. The most recent survey including sitting time data was the Special Eurobarometer 472 (Wave 88.4, December 2017) in 28 countries (30 constituencies) [20, 21]. More information on the Eurobarometer series can be found at http://www.gesis.org/en/eurobarometer/survey-series/standard-special-eb/.

### Data management

From search stages 1–4, each member of the Working Group collated sources of interest into a country-specific Endnote file (or equivalent). Subsequently, the Working Group provided recommendations of the most appropriate data sources using a form (Supplementary file 3) based on the outcomes of interest. Where there was more than one eligible source for a country, recommendations were made based on the following hierarchical criteria:

(i). Sample representativeness: priority was placed on studies where the sampling procedure was intended to provide data representative of the whole country. Samples across geographic areas of a country were preferred to samples restricted to specific areas of a country e.g., a single state or city (termed by the World Health Organisation as “sub-national”).
(ii). Recency: year of data collection.

**Fig. 2:**
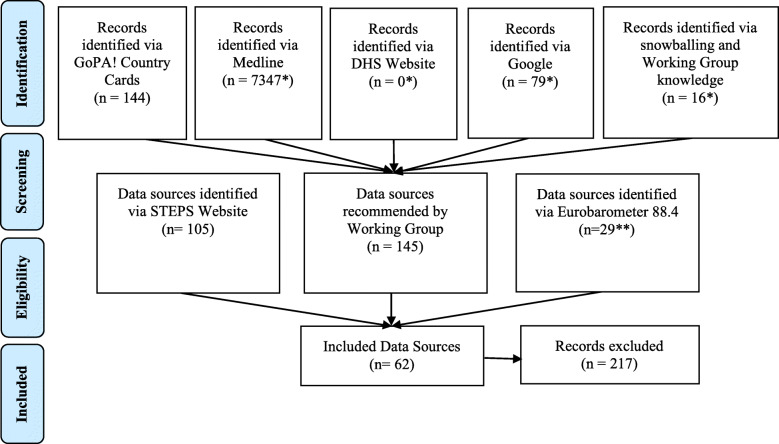
Flowchart of the combined review process

Data sources derived from search strategy stages 5 and 6 were combined with recommendations made by the Working Group. Figure 2 shows the flowchart of the review process.

One researcher (MM) then screened all data sources from stages 5–6 and recommendations from the Working Group for inclusion. A total of 145 data sources were recommended by the working group from stages 1–4. A single data source was selected for each country based on the aforementioned hierarchical criteria.

### Data extraction

Data extraction is described according to each aim.

*(i) What countries have collected country-level data on self-report sitting time in the last 10 years?*

The country name, years of data collection and year of publication were extracted from the data source. The corresponding World Health Organisation region [32], World Bank Income Classification [31] and human population in 2015 [33] were then assigned.

*(ii) What single-item self-report tools have countries used to collect country-level data on sitting time?*

The instrument used to assess self-reported sitting time was extracted from the report.

*(iii) What amount of sitting time is reported according to low-, lower-middle, upper-middle and high-income countries?*

The sample size (n), age range of the sample, mean, standard deviation (SD) and 95% confidence intervals (CI) were extracted from available sources for sitting time. However, a number of data transformations were required due to the unavailability of such data. The full list of data transformations are provided in Supplementary file 4. For Stage 6 of the search, Eurobarometer 88.4 data were downloaded. Firstly, the two constituencies of East and West Germany data were combined. The United Kingdom home nations’ (England, Scotland, Wales and Northern Ireland) data were extracted directly as Great Britain (England, Scotland and Wales) and Northern Ireland respectively. Thus, the total number of possible countries to be searched reduced from 217 to 215.

### Statistical analysis

Data for aims 1 and 2 were extracted directly. For aim 3, sitting time duration was not always readily available, as this outcome was sometimes reported as categorical rather than continuous data. Therefore, we used the midpoint scoring method to estimate the mean and SD [25]. For open-ended categories (e.g. > 6 h) the highest category was truncated at 960 min based on the assumption that the average healthy adult will be awake for at least 16 h per day [19, 34]. For the lowest category (e.g. < 5 h) a lower value of 0 was used. Countries for which this transformation applied included: Saudi Arabia, South Korea and the Eurobarometer 88.4 countries. For studies that reported data separately by sex, a pooled mean estimate and 95% CI were calculated using the *metan* package in STATA. When the median and/or interquartile ranges were presented, recommended formulae were used to transform these values into mean and SD [35].

## Results

A total of 7641 records were identified and screened for eligibility.

*(i) What countries have collected country-level data on self-report sitting time in the last 10 years?*

**Fig. 3:**
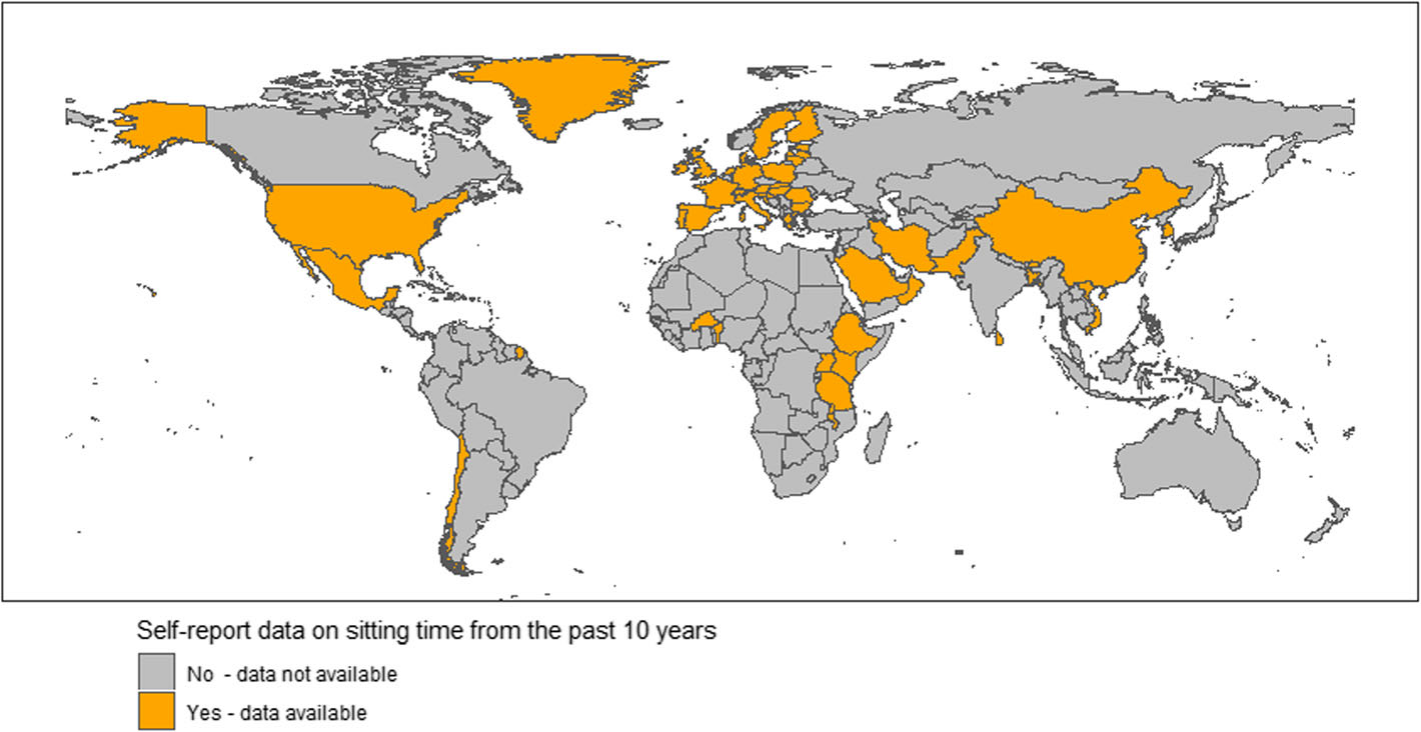
Geographical distribution of countries with a country-level self-report sitting time survey in the last 10 years

Sixty-two countries had eligible data on sitting time (29% of all global countries). These countries represent 47% of the global population in 2015 [31]. The majority of countries were from middle-income (29%) and high-income economies (61%). Data were collected from 2008 to 2012 (*n* = 19) and from 2013 to 2018 (*n* = 43). The findings for each country are listed in Table 1. Half of all countries were from the European region (EURO). Figure 3 shows a map of which countries have collected data. Table 2 summarises the distribution of countries across the World Health Organization geographic regions. Few data sources were identified across South America, Africa and Australasia.

**Table 1:** Country, tool and daily sitting time reported by World Health Organisation Region

Region	Country	World Bank Income Classification^**a**^	Population in 2015 (thousands)	Sample^**c**^	Measurement Method	Sample size	Age (range)	Year of publication	Year(s) of Data Collection	Mean daily sitting time (mins)	Lower 95% CI (mins)	Upper 95% CI (mins)
**AFRO**	Benin	Low	10,576	National	GPAQ	5116	18–69	2016	2015	209	191	228
	Kenya	Lower-middle	47,236	Subnational	GPAQ	5190	18+	2016	2008–2009	203	200	207
	Uganda	Low	40,145	National	GPAQ	3281	18–69	2015	2014	166	158	174
	Burkina Faso	Low	18,111	National	GPAQ	4691	25–64	2014	2013	238	229	247
	Malawi	Low	17,574	National	GPAQ	5204	25–64	2010	2009	157	148	165
	Tanzania (includes Zanzibar)	Low	53,880	National	GPAQ	5762	25–64	2013	2012	132	125	139
	Botswana	Upper-middle	2209	National	GPAQ	4055	15–69	2015	2014	198	187	209
	Ethiopia	Low	99,873	National	GPAQ	9790	15–69	2015	2015	160	154	167
***Total***			*289,604*			*43,089*						
**EMRO**	Qatar	High	2482	National	GPAQ	2496	18–64	2012	2012	245	209	280
	Iran (Iran, Islamic Rep.)	Upper-middle	79,360	National	GPAQ	14,930	15–64	2009	2009	267	259	275
	Saudi Arabia (Combined)	High	31,557	National	GPAQ	9371	15+	2013	2012	333	329	337
	Pakistan	Lower-middle	189,381	National	GPAQ	7358	18–69	2016	2013–2014	223	220	226
	Oman	High	4200	National	GPAQ	2977	18+	2017	2008	225	220	230
	Iraq	Upper-middle	36,116	National	GPAQ	3988	18+	2016	2015–2016	325	308	342
	Kuwait	High	3936	National	GPAQ	3915	18–69	2015	2014	223	218	228
	Lebanon	Upper-middle	5851	Subnational	GPAQ	1973	25–64	2010	2008–2009	587	574	601
***Total***			*352,883*			*47,008*						
**EURO**	Latvia	High	1993	National	IPAQ-short	991	15+	2018	2017	296	287	305
	Italy	High	59,504	National	IPAQ-short	985	15+	2018	2017	302	293	311
	Belgium	High	11,288	National	IPAQ-short	996	15+	2018	2017	308	299	317
	Slovakia (Slovak Republic)	High	5439	National	IPAQ-short	994	15+	2018	2017	314	306	323
	Great Britain	High^b^	65,397	National	IPAQ-short	1008	15+	2018	2017	296	287	305
	France	High	64,457	National	IPAQ-short	1008	15+	2018	2017	287	278	296
	Malta	High	428	National	IPAQ-short	500	15+	2018	2017	278	265	292
	Romania	Upper-middle	19,877	National	IPAQ-short	953	15+	2018	2017	257	246	268
	Slovenia	High	2075	National	IPAQ-short	1035	15+	2018	2017	285	275	294
	Northern Ireland	High^b^	1852	National	IPAQ-short	302	15+	2018	2017	279	264	294
	Switzerland	High	8320	National	IPAQ-long	2730	18–60	2016	2010–2011	366	359	373
	Portugal	High	10,418	National	IPAQ-short	1048	15+	2018	2017	274	265	284
	Spain	High	46,398	National	IPAQ-short	1020	15+	2018	2017	275	267	283
	Croatia	High	4236	National	IPAQ-short	1016	15+	2018	2017	285	275	294
	Netherlands	High	16,938	National	IPAQ-short	1038	15+	2018	2017	394	386	402
	Greenland^d^	High	56	National	IPAQ-long	2122	18+	2017	2014	312	.	.
	Lithuania	High	2932	National	IPAQ-short	1001	15+	2018	2017	292	284	301
	Cyprus (republic of)	High	1161	National	IPAQ-short	500	15+	2018	2017	283	270	297
	Denmark	High	5689	National	IPAQ-short	995	15+	2018	2017	355	347	364
	Austria	High	8679	National	IPAQ-short	984	15+	2018	2017	318	310	326
	Ireland	High	4700	National	IPAQ-short	994	15+	2018	2017	280	271	288
	Poland	High	38,265	National	IPAQ-short	913	15+	2018	2017	282	272	293
	Sweden	High	9764	National	IPAQ-short	1031	15+	2018	2017	346	338	354
	Bulgaria	Upper-middle	7177	National	IPAQ-short	940	15+	2018	2017	325	316	334
	Greece	High	11,218	National	IPAQ-short	1003	15+	2018	2017	338	329	346
	Hungary	High	9784	National	IPAQ-short	997	15+	2018	2017	279	270	288
	Czech Republic	High	10,604	National	IPAQ-short	993	15+	2018	2017	340	330	349
	Estonia	High	1315	National	IPAQ-short	983	15+	2018	2017	328	319	337
	Germany	High	81,708	National	IPAQ-short	1545	15+	2018	2017	304	297	311
	Finland	High	5482	National	IPAQ-short	1009	15+	2018	2017	299	291	308
	Luxembourg	High	567	National	IPAQ-short	494	15+	2018	2017	297	284	310
***Total***			*517,721*			*32,128*						
**PAHO**	Chile	High	17,763	National	GPAQ	5031	18+	2017	2009–2010	171	167	175
	Trinidad/Tobago	High	1360	National	GPAQ	2691	15–64	2012	2010–2011	235	224	246
	Mexico	Upper-middle	125,891	National	IPAQ-short	13,009	20–69	2016	2012	210	210	210
	United States (Combined)	High	319,929	National	IPAQ-short^a^ “similar”	5911	20+	2014	2009–2010	284	278	289
	Virgin Islands	High	135	National	GPAQ	1102	25–64	2010	2009	246	230	261
***Total***			*465,078*			*27,744*						
**SEARO**	Sri Lanka	Upper-middle	20,714	National	GPAQ	5169	18–69	2015	2014–2015	216	205	227
	Maldives	Upper-middle	418	Subnational	GPAQ	1780	15–64	2014	2011	303	292	313
	Bangladesh	Lower-middle	161,201	National	GPAQ	4312	25+	2010	2009–2010	168	164	172
	Bhutan	Lower-middle	787	National	GPAQ	2912	18–69	2015	2014	148	139	157
***Total***			*183,120*			*14,173*						
**WPRO**	Vietnam	Lower-middle	93,572	National	GPAQ	3750	18–69	2016	2015	243	233	253
	Samoa	Upper-middle	194	National	GPAQ	1765	18–64	2014	2013	175	166	185
	Vanuatu	Lower-middle	265	National	GPAQ	4538	25–64	2013	2011–2012	152	143	161
	Tonga	Upper-middle	106	National	GPAQ	2450	25–64	2014	2012	164	157	170
	South Korea (Korea Republic)	High	50,594	National	IPAQ-long	4145	20+	2017	2014	431	425	438
	China	Upper-middle	1,397,029	Subnational	GPAQ	98,424	18+	2012	2010	162	161	163
***Total***			*1,541,760*			*115,072*						

*Abbreviations 95%CI* 95% Confidence Intervals, *IPAQ* International Physical Activity Questionaire, *GPAQ* Global Physical Activity Questionaire, *Mins* minutes, *WPRO* Western Pacific Regional Office, *SEARO* South East Asia Regional Office, *PAHO* Region of the Americas, *EMRO* Eastern Mediterranean Regional Office, *AFRO* African Regional Office, *EURO* European Regional Office

^a^World Bank classifications for country income status for 2020 fiscal year [31]

^b^For Northern Ireland, the Office of National Statistics 2015 population statistic was used. The population of the United Kingdom was used for Great Britain

^c^National samples were those who described a country-wide sampling frame. Sub-national samples were those who selected only certain cities or regions to sample from

^d^Greenland reported no measure of variability (i.e. interquartile range, 95% CI, SD or Standard Error) so only a mean was extracted

**Table 2:** Median of mean sitting times by country income classification

Country Income (World Bank Classification^a^)	Countries (n)	Median of mean sitting times median hours (IQR)
**Low-income**	6	2.7 (2.6–3.3)
**Lower-middle income**	6	3.1 (2.6–3.6)
**Upper-middle income**	12	3.9 (3.2–5.1)
**High-income**^**b**^	38	4.9 (4.7–5.3)
**Total**	62	4.7 (3.5–5.1)

^a^World Bank classifications for country income status for 2020 fiscal year [31]

^b^For Great Britain and Northern Ireland respectively, the World Bank classification used was for the United Kingdom

*(ii) What self-report tools have been used to collect country level data on sitting time?*

Most studies employed the International Physical Activity Questionnaire (IPAQ) (*n* = 34) or the Global Physical Activity Questionnaire (GPAQ) (*n* = 27) to collect data on sitting time [18, 20]. One survey (United States) used an adapted version of the IPAQ (*n* = 1). The IPAQ (both short and long version) uses the single item, *“How much time do you spend sitting on a usual day? This may include time spent at a desk, visiting friends, studying or watching television”*. The GPAQ contains the single item question: *“How much time do you usually spend sitting or reclining on a typical day?”* and this is prefaced by *“The following question is about sitting or reclining at work, at home, getting to and from places, or with friends including time spent sitting at a desk, sitting with friends, traveling in car, bus, train, reading, playing cards or watching television, but do not include time spent sleeping”* [18]. Table 1 outlines the measure used by the countries included in the study.

The Eurobarometer measures sitting time using the IPAQ. The World Health Organisation STEPwise approach to surveillance (STEPS) measures sitting time using the GPAQ [18].

*(iii) What is the duration of sitting time reported by low, lower-middle, upper-middle and high-income countries?*

The median of mean sitting time from all countries (*n* = 62) was 279 min (IQR: 210–304), equivalent to 4.7 h daily. The median of mean sitting times from high-income countries was almost double that of low-income countries (4.9 vs 2.7 h). Table 2 outlines sitting time by World Bank Income classification. World Bank Income Classifications are provided in Supplementary file 5.

## Discussion

This study reviewed all countries globally and collated self-reported country-level data on sitting time. We found just 62 countries (29%) reporting sitting time data in the last 10 years, most of which were high-income countries (61%). Of those countries who did report data, the median of mean daily sitting times was 4.7 (IQR: 3.5–5.1) hours per day. Persons from higher-income countries tended to report sitting longer than those from lower-income countries, with each World Bank Income Classification group from low to high reporting progressively greater sitting times. Most data came from just two sources, the Eurobarometer [25] and the World Health Organisation STEPwise approach to Surveillance (STEPS) Reports [14].

The results of this review should be considered in the context of its limitations. To facilitate comparison between countries, we reduced heterogeneity by restricting to single-item self-report sitting time measures. Such measures have poor accuracy and potentially a lack of validity [16, 22]. Self-report measurement has been found to underreport daily sitting time by 1.4–2.1 h compared with device based measurement [22]. While we used extensive search methods to find relevant data, we expect that some countries have collected data, but have not made these available online. This may be particularly true of countries involved with World Health Organisation STEPwise approach to Surveillance (STEPS) surveillance, who have not made available their reports on the World Health Organisation website. While we were able to seek language translations for STEPWise data (Stage 5), it’s also expected that some countries may have reported data in languages other than English. It was a pragmatic decision to search for studies in English. Limiting to English is consistent with the scoping review process, however its likely data from countries where English is not the first language were missed.

Our study identified data from 62 countries’ from 2008 to 2018, where most data were collected within the latter half of this period. This updates the previous collation of sitting time data from *Rezende* et al. (2016) who collated 54 countries’ data from 2002 to 2011 [9]. The overall sitting time reported in our study and those reported by *Rezende* et al. are similar. Specifically, *Rezende* et al. reported a country population-weighted mean sitting time of 4.7 h per day and a median of mean sitting time of 5 h per day, compared with the present study, which found a median of mean sitting time across all countries of 4.7 h per day [9]. *Rezende* et al.’s sample represented a quarter of the global adult population, whereas the current study represents half of the global adult population (47%). Both studies identified a paucity of data from Africa and Asia, but *Rezende* et al. included older data from South America.

Variations in sitting time across countries were large, ranging from 2.2–9.5 h per day (IQR: 3.5–5.1 h). High-income countries reported sitting almost double that of low-income countries (4.9 vs 2.7 h per day), perhaps because higher-income countries have a higher proportion of the population employed in sedentary occupations [36]. As countries urbanise, and occupations become more sedentary (e.g., greater share of jobs are in service related industries rather than manufacturing/agriculture), it is possible that people in these countries will become more sedentary and sit for longer [36]. In some countries, there may also be underlying social and cultural practices that lead to high sedentary time during leisure [37].

Compared with other risk factors for chronic disease, global coverage of sitting time prevalence data is low (47% of the global population). For example, a recent collation of physical activity data represents 96% of the global population [24] and a collation of smoking prevalence has been generated in 90% of countries [38]. Given the public health impact of high amounts of sitting time [4, 5, 7, 9, 10], the broader global adoption of such sitting time surveillance systems seems warranted. Public health surveillance systems can help inform action, guide public health interventions, evaluate public policy and advocate for policy change [12], which will be required to change and monitor sitting time prevalence. The predominant existing items used in country-level surveillance, the IPAQ and GPAQ, are low-cost and feasible. A stronger global surveillance system will use more accurate measures of sitting time that remain feasible, and have demonstrated sensitivity to change [16, 17, 22, 23]. The Global Observatory for Physical Activity (GoPA!) has begun establishing a physical activity surveillance system that may be a platform to embed sedentary behaviour data collation [30].

## Conclusion

This study provides an updated collation of self-report sitting time globally. The daily sitting time findings should be interpreted cautiously. Sitting time data were collected in 62 of 215 countries, representing 47% of the global adult population. Daily sitting time was on average 4.7 h. There was particularly a lack of data in low- and middle-income countries, but data that were available suggested they reported less daily sitting time than higher-income countries. There is an opportunity to improve surveillance efforts by developing and using improved measures of sitting time and increasing global coverage of countries. Doing so will be crucial to guide future policy, research and practice in managing sedentary behaviour as a risk factor for chronic disease [12], and may be embedded within wider surveillance systems such as The Global Observatory for Physical Activity (GoPA!) [30] and World Health Organisation STEPwise approach to Surveillance (STEPS) [14]. The current data, limited as they are, are being used to inform the second set of Country Cards produced by the Global Observatory for Physical Activity (GoPA!).

## Supplementary information

**Additional file 1: Supplementary file 1.** PRISMA Scoping Review Checklist.

**Additional file 2: Supplementary file 2.** Search Terms used in Stages 2 and 4.

**Additional file 3: Supplementary file 3.** Recommendation Form used by the Working Group.

**Additional file 4: Supplementary file 4.** Transformation formulas used to estimate the mean and standard deviation.

**Additional file 5: Supplementary file 5.** The World Bank country classifications.

## Data Availability

The datasets used during the current study are available within the manuscript in full. Additional queries may be directed to the corresponding author.

## Abbreviations

IPAQ: International Physical Activity Questionnaire
GPAQ: Global Physical Activity Questionnaire
GoPA!: Global Observatory for Physical Activity
SBC: Sedentary Behaviour Council
ISPAH: International Society for Physical Activity and Health
STEPS: STEPwise approach to Surveillance
