# Supplementary material for: Worldwide surveillance of self-reported sitting time: a scoping review
Source: Int J Behav Nutr Phys Act. 2020 Sep 3;17:111. doi: 10.1186/s12966-020-01008-4 (PMC7469304; doi:10.1186/s12966-020-01008-4)
Supplement: Supplementary file 2 — Additional file 2: Supplementary file 2. Search Terms used in Stages 2 and 4. [file 12966_2020_1008_MOESM2_ESM.docx]

**Supplementary File 2**

Search Terms used in Stages 2 and 4.

***Stage 2 Search Terms (PubMED)***

(((((((((((("sedentary behaviour"[All Fields] OR "sedentary behaviours"[All Fields]) OR "sedentary behavior"[All Fields]) OR "sedentary behaviors"[All Fields]) OR "sedentary time"[All Fields]) OR "sitting"[All Fields]) OR "television viewing"[All Fields]) OR "TV viewing"[All Fields]) OR "television time"[All Fields]) OR "TV time"[All Fields]) OR "television watching"[All Fields]) OR "TV watching"[All Fields]) OR "screen time"[All Fields]) OR "computer use"[All Fields] AND ("*country* "[MeSH Terms] OR "*country*"[All Fields]) AND "humans"[MeSH Terms] AND "adult"[MeSH Terms] AND ("2008/XX/XX"[PDat] : "2018/XX/XX"[PDat])

***Stage 4 Search Terms (Google)***

“national survey”, “name of each country” and each of the following in respective searches: “sedentary behaviour”; “sedentary behaviour”; “sedentary time”; “sitting”; “television”; “TV”; “screen”.
