# Supplementary material for: Worldwide surveillance of self-reported sitting time: a scoping review
Source: Int J Behav Nutr Phys Act. 2020 Sep 3;17:111. doi: 10.1186/s12966-020-01008-4 (PMC7469304; doi:10.1186/s12966-020-01008-4)
Supplement: Supplementary file 3 — Additional file 3: Supplementary file 3. Recommendation Form used by the Working Group. [file 12966_2020_1008_MOESM3_ESM.docx]

| **Methods Manual Section Reference** | | **Name of Country:** | | | | Click or tap here to enter text. | | |  |
| --- | --- | --- | --- | --- | --- | --- | --- | --- | --- |
| Section 4.1 | | In the GoPA! Physical Activity Almanac, was physical activity prevalence data for this country sourced from the WHO Global Health Observatory repository? | | | | Choose an item. | | |  |
| Section 4.1 | | If the response to the previous question was “No”, has the GoPA! archive been searched for the alternative source?  If so, what was it? | | | | Choose an item.  Click or tap here to enter text. | | |  |
| Section 4.1  (paragraph 2) | | If the GoPA! archive does not provide any relevant information to find the source, was Google searched for the source? AND  Was the GoPA! Country Card source recommended? | | | | Choose an item. | Choose an item. | | |
| Section 4.2 | | Has the DHS search been performed? AND How many recommended hits?: | | | | Choose an item. | n= | Click or tap here to enter text. | |
| Section 4.2 | | Has the Google search been performed? AND How many recommended hits?: | | | | Choose an item. | n= | Click or tap here to enter text. | |
| Section 4.3 | | Has the PubMed search been performed?  How many hits were identified?  AND How many recommended hits?: | | | | Choose an item. | n= | Click or tap here to enter text. | |
|  |  |  |  |  |  |  | n= | Click or tap here to enter text. | |
| N/A | | Any other sources? If so, what?  E.g. Snowball?: | | | | Click or tap here to enter text. | | |  |
|  | | | | | | | | |  |
| **Recommendations**  **Please order from most recommended to least recommended in the below boxes.**  **Please note, that some countries may have zero recommended sources.**  **Please don’t feel compelled to fill all the boxes below, if for example there is only one obvious recommendation.** | | | | | | | | |  |
| **Indicator 1:**  **Total daily sitting time** | | | | **Indicator 2:**  **Total daily TV viewing** | | | | |  |
| Does this country (potentially) have data relating to this indicator:  *If yes, detail in the boxes below.* | | | No | Does this country (potentially) have data relating to this indicator:  *If yes, detail in the boxes below.* | | | | No |  |
| 1. | Study Name, Authors, Year:  Click or tap here to enter text. | | | 1. | Study Name, Authors, Year:  Click or tap here to enter text. | | | |  |
|  | Is the data available to extract? If so, what data is available (e.g. means (SD) or median (IQR) in h/day)?  Click or tap here to enter text. | | |  | Is the data available to extract? If so, what data is available (e.g. means (SD) or median (IQR) in h/day)?  Click or tap here to enter text. | | | |  |
|  | Link/DOI to data: | | |  | Link/DOI to data:  Click or tap here to enter text. | | | |  |
|  | Notes e.g. age, sample size, any other details:  Click or tap here to enter text. | | |  | Notes e.g. age, sample size, any other details:  Click or tap here to enter text. | | | |  |
| 2. | Study Name, Authors, Year:  Click or tap here to enter text. | | | 2. | Study Name, Authors, Year:  Click or tap here to enter text. | | | |  |
|  | Is the data available to extract? If so, what data is available (e.g. means (SD) or median (IQR) in h/day)?  Click or tap here to enter text. | | |  | Is the data available to extract? If so, what data is available (e.g. means (SD) or median (IQR) in h/day)?  Click or tap here to enter text. | | | |  |
|  | Link/DOI to data:  Click or tap here to enter text. | | |  | Link/DOI to data:  Click or tap here to enter text. | | | |  |
|  | Notes e.g. age, sample size, any other details:  Click or tap here to enter text. | | |  | Notes e.g. age, sample size, any other details:  Click or tap here to enter text. | | | |  |
| 3. | Study Name, Authors, Year:  Click or tap here to enter text. | | | 3. | Study Name, Authors, Year:  Click or tap here to enter text. | | | |  |
|  | Is the data available to extract? If so, what data is available (e.g. means (SD) or median (IQR) in h/day)?  Click or tap here to enter text. | | |  | Is the data available to extract? If so, what data is available (e.g. means (SD) or median (IQR) in h/day)?  Click or tap here to enter text. | | | |  |
|  | Link/DOI to data:  Click or tap here to enter text. | | |  | Link/DOI to data:  Click or tap here to enter text. | | | |  |
|  | Notes e.g. age, sample size, any other details:  Click or tap here to enter text. | | |  | Notes e.g. age, sample size, any other details:  Click or tap here to enter text. | | | |  |
| 4. | Study Name, Authors, Year:  Click or tap here to enter text. | | | 4. | Study Name, Authors, Year:  Click or tap here to enter text. | | | |  |
|  | Is the data available to extract? If so, what data is available (e.g. means (SD) or median (IQR) in h/day)?  Click or tap here to enter text. | | |  | Is the data available to extract? If so, what data is available (e.g. means (SD) or median (IQR) in h/day)?  Click or tap here to enter text. | | | |  |
|  | Link/DOI to data:  Click or tap here to enter text. | | |  | Link/DOI to data:  Click or tap here to enter text. | | | |  |
|  | Notes e.g. age, sample size, any other details:  Click or tap here to enter text. | | |  | Notes e.g. age, sample size, any other details:  Click or tap here to enter text. | | | |  |
|  |  | | |  |  | | | |  |
| **FINAL RECOMMENDATION**  For your top recommendation for total daily sitting time and total daily TV viewing time, ranked above as number 1, please provide a brief rationale in the boxes below, based on the following, hierarchical, criteria:     1. **Representativeness**: does the manuscript indicate that sampling procedures intended to provide national population-representative data: yes/no. If no, how representative is it? 2. **Measurement**: does the manuscript provide any info on measurement properties/methodological development of the sedentary behaviour measure: yes/no. If yes, briefly provide details (e.g. “WHO questionnaire which followed a standardised translation and cultural adaptation process and was pilot tested and validated in the target population”; “Actigraph accelerometer worn on the right hip”) 3. **Recency:** Year(s) of data collection (e.g. 2016); Was the recommended survey the most recent one of its kind: yes/no | | | | | | | | |  |
| **Total daily sitting time (indicator 1):**  Representativeness:  Choose an item.  Click or tap here to enter text.  Measurement:  Choose an item.  Click or tap here to enter text.  Recency:  Click or tap here to enter text.  Choose an item. | | | | **Total daily TV viewing (indicator 2):**  Representativeness:  Choose an item.  Click or tap here to enter text.  Measurement:  Choose an item.  Click or tap here to enter text.  Recency:  Click or tap here to enter text.  Choose an item. | | | | |  |
|  | | | | | | | | |  |
| **Please now also complete the form for National sedentary behaviour surveillance (indicator 3).**  **This form can be found at:**  [**https://drive.google.com/drive/folders/1htv9bHH_Fha5n1LJQbr5fAqSvqa1zDwh?usp=sharingLINK**](https://drive.google.com/drive/folders/1htv9bHH_Fha5n1LJQbr5fAqSvqa1zDwh?usp=sharingLINK) | | | | | | | | |  |
| **Once both this form, and the form for indicator 3 are complete for a particular country, please email them to Tepi Mclaughlin and Andy Atkin**  [**Matthewmclaughlinemail@gmail.com**](mailto:Matthewmclaughlinemail@gmail.com) **\|** [**A.Atkin@uea.ac.uk**](mailto:A.Atkin@uea.ac.uk) | | | | | | | | |  |
