# Supplementary material for: Worldwide surveillance of self-reported sitting time: a scoping review
Source: Int J Behav Nutr Phys Act. 2020 Sep 3;17:111. doi: 10.1186/s12966-020-01008-4 (PMC7469304; doi:10.1186/s12966-020-01008-4)
Supplement: Supplementary file 4 — Additional file 4: Supplementary file 4. Transformation formulas used to estimate the mean and standard deviation. [file 12966_2020_1008_MOESM4_ESM.docx]

**Supplementary File 4**

**Transformation formulas used to estimate the mean and standard deviation**

| **Description of transformation** | **Transformation formula used** | **Countries transformation applied to** |
| --- | --- | --- |
| Mean from categorical data | $\frac{\sum xf}{n}$ | Saudi Arabia  South Korea |
| Standard deviation from categorical data | $\sqrt{\frac{\sum(x-\bar{x})^{2}f}{n-1}}$ | Saudi Arabia  South Korea |
| Mean from median and interquartile 1 and 3 (25) | $\frac{w(q_{1}+q_{3})}{2}+\left( 1-w \right)m$ | Chile |
| Standard deviation from interquartile 1 and 3 (26). | $\frac{(q_{1}-q_{3})}{\eta(n)}$ | Chile  Bangladesh |
| Standard deviation from interquartile range | $\frac{IQR}{1.35}$ | Pakistan  Oman |
| Where:  $x=Category midpoint$  $f=Number in category$  $n=sample size$  $w=weight assigned following Lou et al ADDIN EN.CITE <EndNote><Cite><Author>Lou</Author><Year>2017</Year><RecNum>2459</RecNum><IDText>Optimally estimating the sample mean from the sample size, median, mid-range, and/or mid-quartile range</IDText><DisplayText>(25)</DisplayText><record><rec-number>2459</rec-number><foreign-keys><key app="EN" db-id="xvzav92e322dprevxdipfaazzrpezzrwaz2r" timestamp="1579659288">2459</key></foreign-keys><ref-type name="Journal Article">17</ref-type><contributors><authors><author>Lou, D. et al.</author></authors></contributors><titles><title>Optimally estimating the sample mean from the sample size, median, mid-range, and/or mid-quartile range</title><secondary-title>Statistical Methods in Medical Research</secondary-title></titles><periodical><full-title>Statistical Methods in Medical Research</full-title></periodical><pages>1785-1805</pages><volume>27</volume><number>6</number><dates><year>2017</year></dates><urls></urls></record></Cite></EndNote>(25)$  $q_{1}=Interquartile 1$  $q_{3}=Interquartile 3$  $m=median$  $\eta\left( n \right)=Functionof n following Wan et al ADDIN EN.CITE <EndNote><Cite><Author>Wan</Author><Year>2014</Year><RecNum>2460</RecNum><IDText>Estimating the sample mean and standard deviation from the sample size, median, range and/or interquartile range.</IDText><DisplayText>(26)</DisplayText><record><rec-number>2460</rec-number><foreign-keys><key app="EN" db-id="xvzav92e322dprevxdipfaazzrpezzrwaz2r" timestamp="1579659288">2460</key></foreign-keys><ref-type name="Journal Article">17</ref-type><contributors><authors><author>Wan, X. et al</author></authors></contributors><titles><title>Estimating the sample mean and standard deviation from the sample size, median, range and/or interquartile range.</title><secondary-title>BMC Medical Research Methodology</secondary-title></titles><periodical><full-title>BMC Med Res Methodol</full-title><abbr-1>BMC medical research methodology</abbr-1></periodical><dates><year>2014</year></dates><urls></urls></record></Cite></EndNote>(26).$  $IQR=Interquartile range$ | | |
