# Supplementary material for: Worldwide surveillance of self-reported sitting time: a scoping review
Source: Int J Behav Nutr Phys Act. 2020 Sep 3;17:111. doi: 10.1186/s12966-020-01008-4 (PMC7469304; doi:10.1186/s12966-020-01008-4)
Supplement: Supplementary file 5 — Additional file 5: Supplementary file 5. The World Bank country classifications. [file 12966_2020_1008_MOESM5_ESM.docx]

**Supplementary File 5**

The World Bank country classifications.

| **Classification as per World Bank 2020 Fiscal year(1).** | **Definition** |
| --- | --- |
| Low income | Low income economies are those with GNI per capita, calculated using the *World Bank Atlas method*, of $1,025 or less in 2018. |
| Lower-middle income | Lower-middle-income economies are those with a GNI per capita, calculated using the *World Bank Atlas method*, of more than $1,025 but less than $3,995 |
| Upper-middle income | Upper-middle-income economies those with a GNI per capita, calculated using the *World Bank Atlas method*, of more than $3,995 but less than $12,375 |
| High-income | High-income economies are those with a GNI per capita, calculated using the *World Bank Atlas method*, of $12,376 or more. |

References

1. Bank TW. World Bank Country and Lending Groups 2019 [Accessed: 12/08/2019; Available via: <https://datahelpdesk.worldbank.org/knowledgebase/articles/906519>.
